# Assessment of Risk for Recurrent Diverticulitis: A Proposal of Risk Score for Complicated Recurrence

**DOI:** 10.1097/MD.0000000000000557

**Published:** 2015-02-27

**Authors:** Ville Sallinen, Juha Mali, Ari Leppäniemi, Panu Mentula

**Affiliations:** From the Department of Abdominal Surgery, University of Helsinki, Helsinki University Hospital, Helsinki, Finland.

## Abstract

Recurrence of acute diverticulitis is common, and—especially complicated recurrence—causes significant morbidity. To prevent recurrence, selected patients have been offered prophylactic sigmoid resection. However, as there is no tool to predict whose diverticulitis will recur and, in particular, who will have complicated recurrence, the indications for sigmoid resections have been variable. The objective of this study was to identify risk factors predicting recurrence of acute diverticulitis.

This is a retrospective cohort study of patients presenting with computed tomography–confirmed acute diverticulitis and treated nonresectionally during 2006 to 2010. Risk factors for recurrence were identified using uni- and multivariate Cox regression.

A total of 512 patients were included. History of diverticulitis was an independent risk factor predicting uncomplicated recurrence of diverticulitis (1–2 earlier diverticulitis HR 1.6, 3 or more—HR 3.2). History of diverticulitis (HR 3.3), abscess (HR 6.2), and corticosteroid medication (HR 16.1) were independent risk factors for complicated recurrence. Based on regression coefficients, risk scoring was created: 1 point for history of diverticulitis, 2 points for abscess, and 3 points for corticosteroid medication. The risk score was unable to predict uncomplicated recurrence (AUC 0.48), but was able to predict complicated recurrence (AUC 0.80). Patients were further divided into low-risk (0–2 points) and high-risk (>2 points) groups. Low-risk and high-risk groups had 3% and 43% 5-year complicated recurrence rates, respectively.

Risk for complicated recurrence of acute diverticulitis can be assessed using risk scoring. The risk for uncomplicated recurrence increases along with increasing number of previous diverticulitis.

## INTRODUCTION

Acute diverticulitis is a common disease with a tendency to recur.^1^ Stage of the acute diverticulitis will determine the morbidity and mortality involved in acute diverticulitis with lower stages having a rather benign course and higher stages having up to 32% mortality.^2^ Patients have been offered prophylactic sigmoid resection in order to prevent recurrence of acute diverticulitis and the concomitant morbidity associated with the higher stages.^3^ However, the indications for elective sigmoid resection have been highly variable. In history, two acute diverticulitis—or even one on a young patient—were considered an indication for a sigmoid resection.^4,5^ Recent evidence has shown, however, that the first diverticulitis is usually the most severe one, and the recurrent attacks are usually uncomplicated.^6,7^ This has been thought to be due to increased scar tissue around the diverticula, which would prevent or limit the perforation. Yet, small portion of patients will develop complicated recurrence,^8^ and prediction and prevention of that event are of paramount importance. Earlier studies have provided risk factors for recurrence,^4,5,8,9^ but clinical decision making has been difficult nevertheless. Current guidelines are unable to make strong recommendations for patient selection for prophylactic sigmoid resection.^10^

Aims of this study was to (1) identify independent risk factors predicting complicated recurrence of acute diverticulitis, and (2) form a simple score to be used in bed-side clinical setting to determine which patients are at high risk for complicated recurrence.

## METHODS

This was a retrospective cohort study conducted at an academic teaching hospital that functions as both a secondary referral center and tertiary referral center serving a population of approximately 1.5 million. Data were gathered from a database established earlier, which comprise of all patient admissions due to acute colonic diverticulitis from 2006 to 2010.^11^ A total of 968 patient admissions were identified from the database.

Patients without computed tomography (CT) verification were also excluded (n = 335) because clinical diagnostic accuracy is poor.^12^ Patients with obstruction (n = 17) or colon cancer mimicking diverticulitis (n = 17) were excluded. Further, patients who underwent emergency colonic resection were excluded (n = 76) because these patients are not at risk for recurrence. Readmissions within 30 days (n = 11) were considered an activation of the primary acute diverticulitis, and these were incorporated into index admissions.

Data regarding patient demographics, comorbidities, medications, prior abdominal operations, history of acute diverticulitis, CT-scan findings at index admission, laboratory parameters at index admission, treatment at index admission, classification of acute diverticulitis at index admission, elective sigmoid resection during follow-up, as well as incidence and type of recurrence during follow-up were extracted.

Uncomplicated diverticulitis was defined as radiological signs of acute diverticulitis (thickened bowel wall segment, inflammation in pericolonic fat, and/or pericolic air bubbles) without distant intra- or retroperitoneal air, abscess, fistula, obstruction, or peritonitis (Stage 1 diverticulitis).^2^ Complicated diverticulitis is defined as radiological signs of acute diverticulitis with an abscess, fistula, obstruction, peritonitis, large amount of fluid in the abdominal cavity, and/or distant intra- or retroperitoneal air (Stage 2 or higher).^2^ Distribution of extraluminal air has been defined earlier, and showed to have an effect on treatment and morbidity.^13^

Recurrence of diverticulitis was defined as a CT-verified diverticulitis or clinical diverticulitis (fever, elevated C-reactive protein levels, left lower abdominal pain) with treatment (per oral or intravenous antibiotics). Recurrences were diagnosed and treated both as in- and outpatient and at the hospital and general practitioners visits. To gather follow-up data, electronical patient records were analyzed from our hospital, as well as from the family practitioners/health care centers at the Helsinki area. Follow-up was determined as the time from discharge to the examination of the patient records.

Statistical analyses were made using SPSS Statistics v. 21 (IBM, Armonk, NY). Cox univariate regression was used for univariate analyses. Continuous variables that had *P* < 0.20 were further modified to obtain categorical variables, which could be inserted into multivariate analyses. Variables that had *P* < 0.20 were considered for multivariate analysis. If variables were multicollinear, one that was most easily accessible in terms of clinical use and/or showed highest hazard ratio was selected. Multivariate analyses were performed using Cox regression analysis with forward likelihood ratio method. Kaplan–Meier with log-rank test was used to analyze recurrence-free time, with sensoring for death, loss of follow-up, or elective sigmoid resection. Appropriate permissions to conduct the study were obtained from the hospital and Helsinki health care centers’ institutional review boards.

## RESULTS

### Patients

The cohort consisted of 512 patients with CT-diagnosed acute colonic diverticulitis. Basic patient demographics are shown in Table 1.

**TABLE 1:**
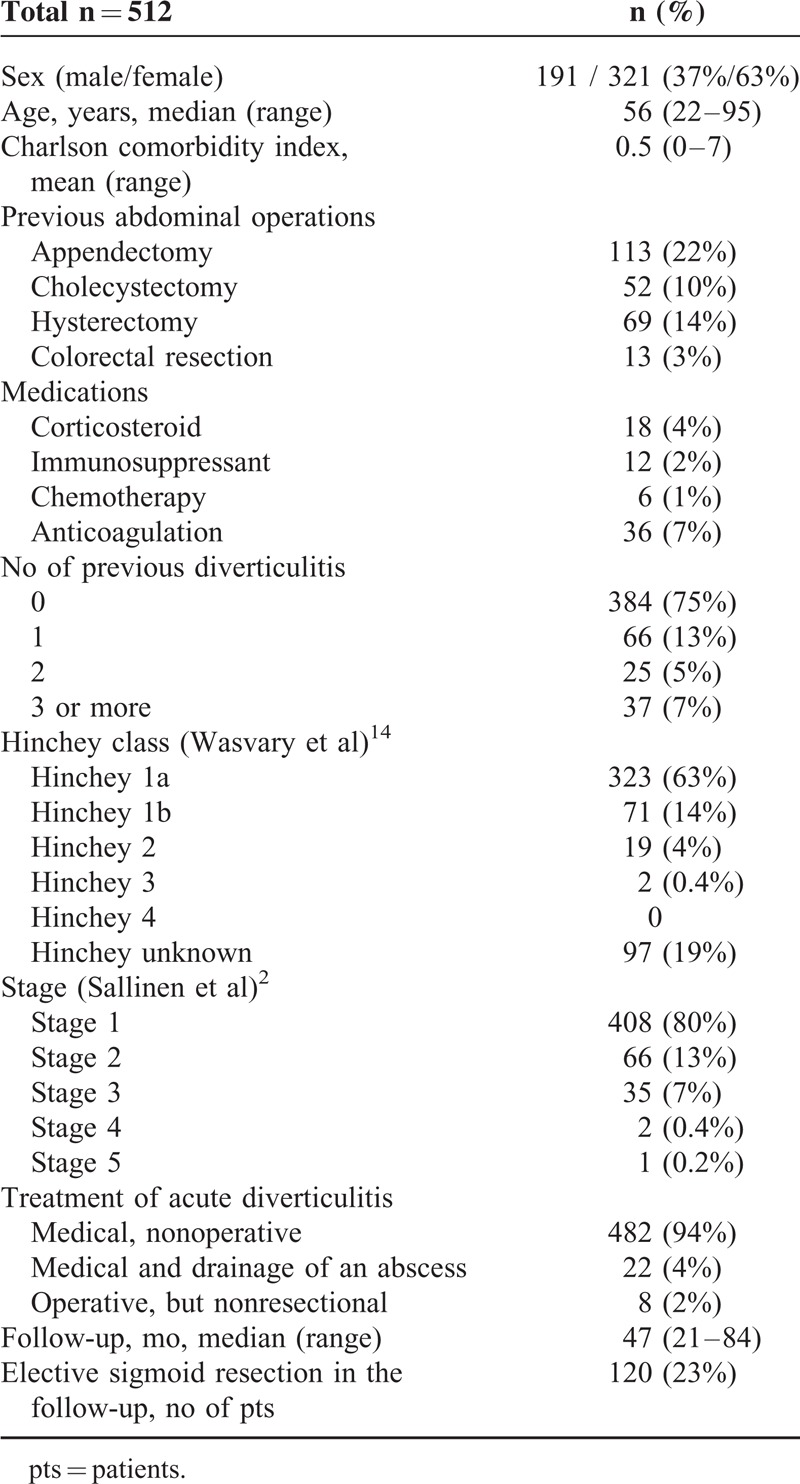
Basic Demographics of Patients Included in the Study

### Univariate Analysis

A univariate analysis was performed for age, sex, comorbidities, earlier operations, medication, laboratory parameters on index admission, history of diverticulitis, and CT findings to detect risk factors for recurrent uncomplicated or complicated diverticulitis (Table 2). History of diverticulitis (HR 2.0–3.0) and history of complicated diverticulitis (HR 2.6) were the strongest risk factor for recurrence of uncomplicated diverticulitis (HR 3.1). Age over 75 years (HR 3.3), Charlson comorbidity index over 1 (HR 2.8), corticosteroid use (HR 15.4), anticoagulation (HR 3.9), history of any (HR 2.7) or complicated diverticulitis (HR 4.9), complicated index diverticulitis (HR 6.6), white blood cell count over 10 × 10^9^/L on index diverticulitis (HR 8.5), distant retroperitoneal air (HR 11.1), and abscess (HR 6.3) were the strongest risk factors for recurrence of complicated acute diverticulitis (Table 2).

**TABLE 2:**
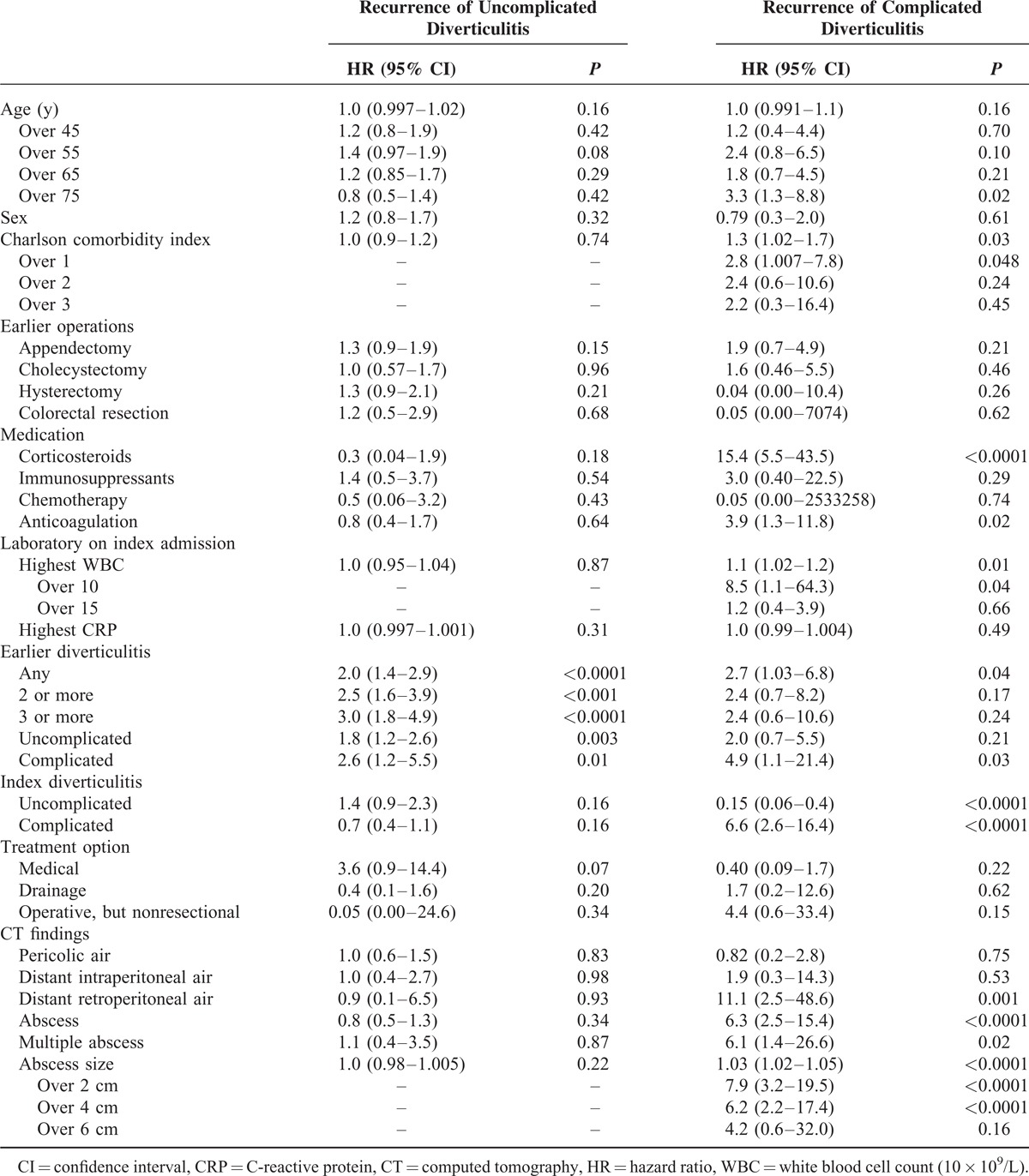
Univariate Analysis of Risk Factors Associated With Uncomplicated and Complicated Recurrence of Acute Diverticulitis

### Multivariate Analysis

Multivariate analysis identified history of diverticulitis (1–2 earlier diverticulitis—HR 1.6, 3 or more—HR 3.2) as an independent risk factor for recurrence of uncomplicated acute diverticulitis (Table 3). On the other hand, history of any diverticulitis (HR 3.3), abscess (HR 6.2), and corticosteroid medication (HR 16.1) were identified as independent risk factors for complicated recurrence of acute diverticulitis (Table 4).

**TABLE 3:**
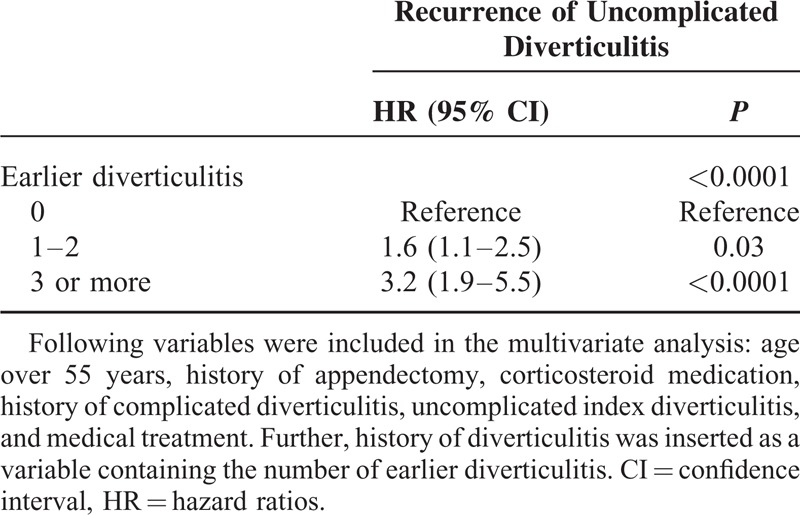
Multivariate Analysis of Risk Factors Predicting Uncomplicated Recurrence

**TABLE 4:**
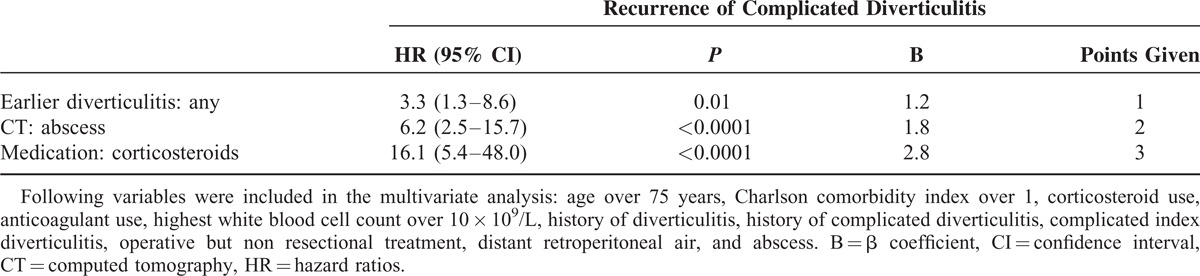
Multivariate Analysis of Risk Factors Predicting Complicated Recurrence

### Formation of Risk Score for Complicated Recurrence

Regression (β) coefficients for independent risk factors predicting complicated recurrence of acute diverticulitis were 1.2 for history of diverticulitis, 1.8 for an abscess, and 2.8 for corticosteroid medication (Table 4). To obtain risk score points, these figures were rounded to nearest integer, yielding 1 point for history of diverticulitis, 2 points for an abscess, and 3 points for corticosteroid medication (Table 4).

### Evaluation of the Risk Score Performance and Division to Low-Risk and High-Risk Groups

**FIGURE 1:**
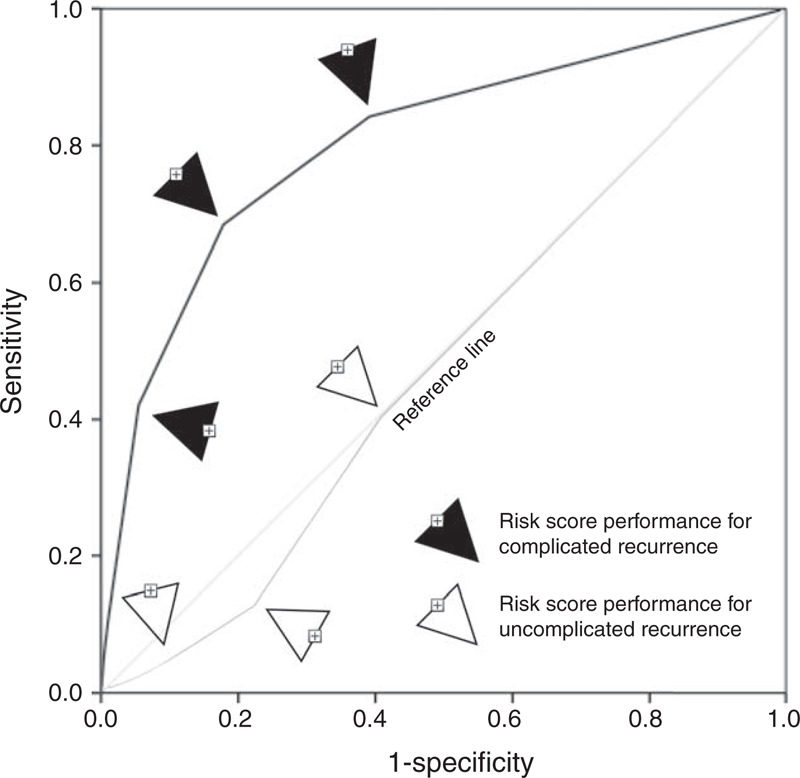
ROC curves showing the predictive ability of the risk score. Notice that the risk score is poor predictor of uncomplicated recurrence (AUC 0.48, SE 0.03, 95% confidence interval 0.42–0.53, *P* = 0.42), but shows ability to predict complicated recurrence (AUC 0.80, SE 0.06, 95% confidence interval 0.69–0.92, *P* < 0.0001).

The risk score performed well in predicting complicated recurrence (AUC 0.80, SE 0.06, 95% confidence interval 0.69–0.92, *P* < 0.0001), but not in predicting uncomplicated recurrence (AUC 0.48, SE 0.03, 95% confidence interval 0.42–0.53, *P* = 0.42) (Figure 1). Cutoff for low risk versus high risk was determined by the point in ROC curve, in which positive and negative predictive values were highest. This point was at risk score of ≥3 points. Accordingly, patients with risk score 0 to 2 points were classified as low risk, and patients with more than 2 points (3–6 points) were classified as high risk.

### Prognosis in Low-Risk Versus High-Risk Groups

**FIGURE 2:**
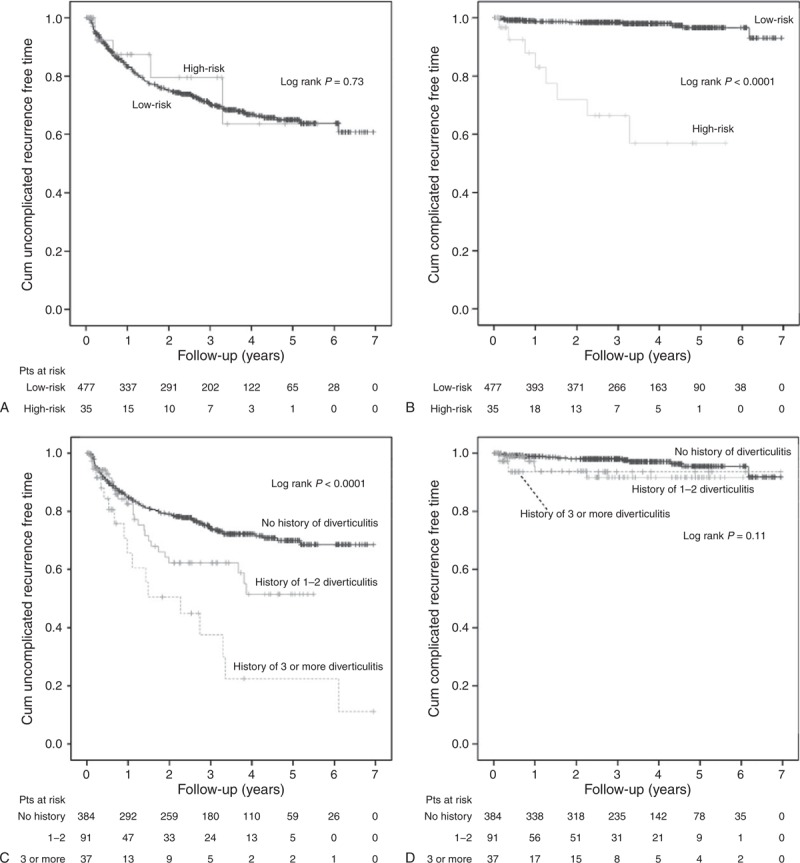
Kaplan–Meier survival curves. (A) Cumulative uncomplicated recurrence-free time of low-risk (0–2 points) versus high-risk (>2 points) patients. Sensoring for loss of follow-up, death, or sigmoid resection. (B) Cumulative complicated recurrence-free time of low-risk versus high-risk patients. Sensoring for loss of follow-up, death, or sigmoid resection. (C) Cumulative uncomplicated recurrence-free time of patients with or without history of diverticulitis. Sensoring for loss of follow-up, complicated recurrence, death, or sigmoid resection. (D) Cumulative complicated recurrence-free time of patients with or without history of diverticulitis. Sensoring for loss of follow-up, death, or sigmoid resection. pts = patients.

Low-risk versus high-risk groups showed statistically significant cumulative complicated recurrence-free time in Kaplan–Meier survival analysis (Figure 2B). However, no difference was noted in low-risk versus high-risk groups in regard to uncomplicated recurrence (Figure 2A). Five-year complicated, but not uncomplicated, recurrence-free rate was higher in low-risk group (Table 5).

**TABLE 5:**
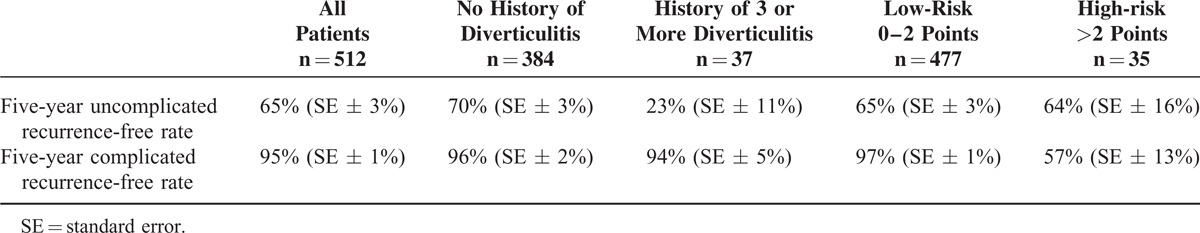
Five-Year Recurrence-Free Rates in Patients With No History Versus History of 3 or More Diverticulitis and Low-Risk Versus High-Risk for Recurrence (Kaplan–Meier Estimate)

### Relationship of History of Diverticulitis on Future Recurrence

As history of diverticulitis was the only independent risk factor for uncomplicated recurrence, Kaplan–Meier survival curves were created to analyze the relationship of recurrence in regard to history of diverticulitis (Figure 2C and D). History of diverticulitis as a single factor was a prognostic factor for uncomplicated, but not complicated, recurrence of acute diverticulitis (Figure 2C and D). Patients with 3 or more earlier diverticulitis had 23% 5-year uncomplicated recurrence-free rate compared to 70% recurrence-free rate of patients without earlier diverticulitis (Table 5). History of earlier diverticulitis as a single factor did not have an effect on 5-year complicated recurrence-free rate (96% vs 94%) (Table 5).

## DISCUSSION

We report here that the risk factors for uncomplicated and complicated recurrence of acute diverticulitis are different. We provide a simple risk score with grouping into low-risk and high-risk patient populations to predict recurrence of complicated diverticulitis. The risk score consists of 3 simple parameters (history of diverticulitis, abscess, corticosteroid medication), which are easily assessable bed-side during ward rounds. Further, we show that patients with a history of 3 or more diverticulitis are at greatest risk for uncomplicated recurrence.

Several earlier studies have analyzed risk factors for overall and complicated recurrence of acute diverticulitis. Risk factors such as C-reactive protein,^15^ age over 50 years,^5^ age less than 50 years,^4^ Charlson comorbidity index,^5^ history of diverticulitis,^4^ family history of diverticulitis,^8^ male sex,^16^ corticosteroid use,^17^ and retroperitoneal abscess^8^ have been associated with recurrence with variable risk. Nevertheless, clinical decision making regarding prophylactic elective sigmoid resection has been difficult.^10^ A few reports have shown increased risk of complicated recurrence in patients using corticosteroid medication^18–20^ and prophylactic sigmoid resection is probably warranted in these patients with lower threshold,^1^ while also bearing in mind that comorbidities in these patients might rule out major surgical procedure.^21^ On the other hand, patients with corticosteroid medication are at higher risk for mortality due to complicated diverticulitis, and complicated recurrence might be fatal.^22^

Patients who have a history of diverticulitis, particularly with a history of three or more, are at great risk for uncomplicated recurrence. However, several former diverticulitis does not seem to increase the risk for complicated recurrence. If several recurrent diverticulitis is used as the only indication for prophylactic sigmoid resection, one must bear in mind that the operation will lower uncomplicated recurrence rate, but might not be effective in preventing complicated recurrence. Our 5-year overall (39%) and complicated (5%) recurrence rates were highly similar to those reported by others (36% and 3.9%).^8^ Thus, complicated recurrence rate is low, and careful patient selection is needed to prevent complicated recurrence. The risk score described here can pick up high-risk patients (7% of patients), of whom 66% will have recurrent diverticulitis and 43% will develop complicated recurrence in 5-year follow-up. Further, patients in low-risk group (93% of patients) will have 5-year complicated recurrence-free rate of 97%.

This study has limitations. First, this was a single-center retrospective cohort with all its inherited limitations and the external validity is thus limited. Second, follow-up was done by examining the hospital's and health care centers’ electronical patient records, and patients were not contacted. This may lead to underestimation of the recurrence rate as patients may have sought treatment from private clinics. However, public health care in Finland remains popular, and we estimate that the majority of patients have used public services to seek treatment. Third, 23% of patients underwent an elective sigmoid resection in the follow-up. As sigmoid resection will prevent majority of recurrences, this has a major impact on the recurrence rates. This is taken into account in the Cox regression analyses and Kaplan–Meier survival curves with sensoring the cases at the time of sigmoid resection, but still natural recurrence rates are likely underestimated. Fourth, there is a limited number of patients who developed a complicated recurrence in the follow-up (26 out of 512 patients at 5-year follow-up), which merely shows that complicated recurrence is rare.

In conclusion, the risk score provides an easy and straightforward tool to predict complicated recurrence of diverticulitis. Uncomplicated recurrence is likely if a patient has already had three or more diverticulitis before.
